# Applications of *Pythium*- and *Phytophthora*-produced volatiles in plant disease control

**DOI:** 10.1007/s00253-024-13312-1

**Published:** 2024-10-03

**Authors:** Taha Majid Mahmood Sheikh, Jinhao Chen, Lunji Wang, Dongmei Zhou, Sheng Deng, Juliana Velasco de Castro Oliveira, Waseem Raza, Lihui Wei, Paul Daly

**Affiliations:** 1 Key Lab of Food Quality and Safety of Jiangsu Province—State Key Laboratory Breeding Base, Institute of Plant Protection, Jiangsu Academy of Agricultural Sciences, 50 Zhongling St, Nanjing, 210014 China; 2 Department of Cell Biology and Genetics, Shantou University Medical College, Shantou, China; 3 College of Food and Bioengineering, Henan University of Science and Technology, Luoyang, Henan China; 4 SENAI Innovation Institute for Biotechnology, São Paulo, Brazil; 5 Jiangsu Provincial Key Lab for Organic Solid Waste Utilization, National Engineering Research Center for Organic-Based Fertilizers, Jiangsu Collaborative Innovation Center for Solid Organic Waste Resource Utilization, Nanjing Agricultural University, Nanjing, China

**Keywords:** Volatile, *Pythium*, *Phytophthora*, Disease detection, Biocontrol

## Abstract

**Abstract:**

Volatile organic compounds (VOCs) mediate biological interactions and are produced by *Pythium* and *Phytophthora* species. These VOCs are biotechnologically relevant because the genera include important plant pathogens, whereby VOCs can aid in disease detection, and biological control agents, whereby VOCs contribute to disease control. Studies on VOC production, identification, and characterization of individual VOCs produced by *Pythium* and *Phytophthora* species are reviewed. VOCs detected in plants infected with *Phytophthora* species are also reviewed as potentially oomycete-derived VOCs. The *Pythium-* and *Phytophthora*-produced VOCs are compared with other microorganisms, and the main effects of these VOCs on microbial inhibition and plant-mediated effects are reviewed. These effects are summarized from direct demonstration studies and inferences based on the known functions of the identified *Pythium-* and *Phytophthora*-produced VOCs. There are two main applications of VOCs to plant disease control: the use of VOCs to detect pathogenic *Pythium* and *Phytophthora* species, e.g., e-nose detecting systems, and the use of VOC-producing biological control agents, e.g., *Pythium oligandrum*. Future research could understand how the VOCs are produced to engineer VOC levels in strains, analyze more oomycete species and strains, accurately quantify the VOCs produced, and exploit recent developments in analytical chemistry technology.

**Key points:**

•* Compiled inventory of volatiles produced by Phytophthora and Pythium species*

•* Volatilomes contain microbe-inhibiting and plant growth-promoting compounds*

•* Volatile potential in disease detection and control supports analyzing more species*

**Supplementary Information:**

The online version contains supplementary material available at 10.1007/s00253-024-13312-1.

## Introduction to volatile organic compounds (VOCs) and their importance

Volatile organic compounds (VOCs) are low molecular organic weight compounds with low boiling points, high vapor pressure, and lipophilic properties. The *Pythium* and *Phytophthora* genera are two of the most important genera of oomycetes. *Phytophthora* and *Pythium* species include several of the most devastating plant pathogens (Kamoun et al. 2015), and the *Pythium* genus also includes important antagonists that can be applied as biological control agents (Ho 2018). *Pythium*- and *Phytophthora-*produced VOCs are important in both microbe-microbe and plant–microbe interactions where the VOCs can inhibit the growth of other microbes and have either beneficial or detrimental effects on plants. In terms of biotechnological applications for agriculture, the VOCs produced by *Pythium* biocontrol agents could form part of an environmentally sustainable alternative to conventional pesticides (Tilocca et al. 2020), and the *Phytophthora*-produced VOCs can aid in the detection of *Phytophthora*-caused diseases (MacDougall et al. 2022).

**Fig. 1:**
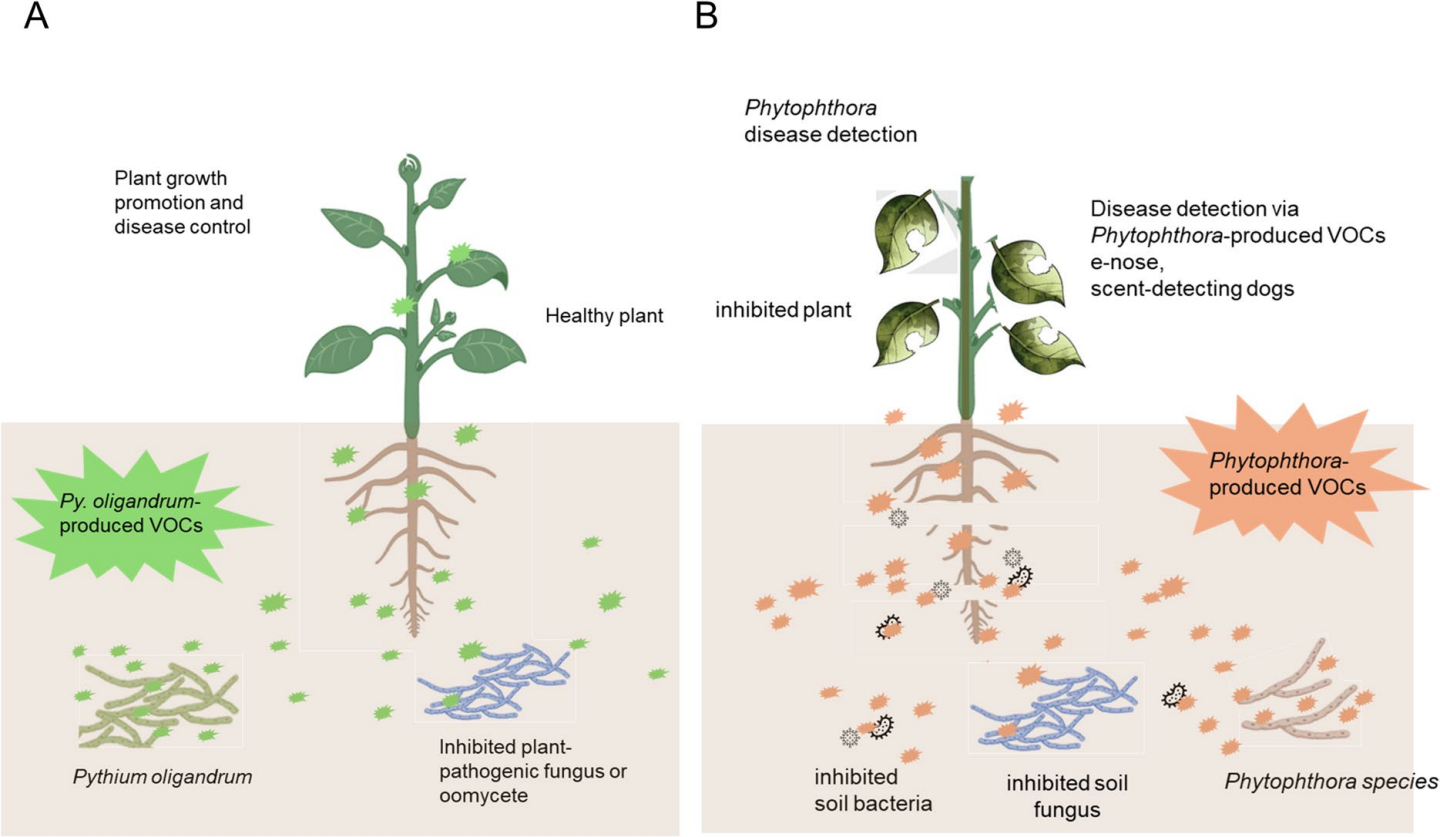
Schematic of the effects and potential applications of **A** volatile organic compounds produced from beneficial *Pythium* species such as *Py. oligandrum* and **B** volatile organic compounds produced by plant-pathogenic *Phytophthora* species on plants and other microorganisms. Parts of the images were obtained from BioRender.com

Previously, reviews have summarized fungal (El Jaddaoui et al. 2023) or both fungal and bacterial-produced VOCs (Almeida et al. 2022) and their potential for plant protection via microbial inhibition, as well as other plant protection mechanisms of bacterial-produced VOCs (Rani et al. 2023) or have covered the relationship of plant health with bacterial volatiles (Garbeva and Weisskopf 2020) or relationship of plant health with both fungal and bacterial VOCs (Poveda 2021). In previous reviews on microbial volatile compounds, oomycetes are almost always the target of the VOCs, not the source, or VOCs are attributed to the plant infected by an oomycete plant pathogen. Very recently, VOCs and their contribution to plant defense were reviewed, including oomycete VOCs produced by the plant-beneficial *Pythium oligandrum* species (Montejano-Ramírez et al. 2024). Here, we review VOCs produced by oomycetes from *Pythium* and *Phytophthora* genera and their applications in plant disease control as overviewed schematically in Fig. 1.

## Volatile production by *Pythium* and *Phytophthora* species

### Production of volatile compounds and identification of produced VOCs

Several older and more recent studies describe the production of VOCs by *Pythium* or *Phytophthora* species, although no subsequent identification of the VOCs was done. *Py. oligandrum* produced volatile compounds that inhibited the growth of *Ascochyta medicaginicola* (syn. *Phoma medicaginis*) (Bradshaw-Smith et al. 1991), and *Fusarium oxysporum* and *Py. ultimum* (El-Katatny et al. 2006). More recently, volatile compounds from *Py. oligandrum* reduced *Fusarium graminearum* mycotoxin production (Pellan et al. 2021). E-nose technology has been used to detect volatile compounds from *Ph. plurivora* (Borowik et al. 2022), and *Py. intermedium* (Borowik et al. 2021). In contrast to these reports detecting the production of volatile compounds, there was a report of the lack of detection of *Py. aphanidermatum* volatile compounds (Sánchez-Fernández et al. 2016).

From the literature, 84 VOCs have been identified from monocultures of one *Pythium* species and 10 *Phytophthora* species (Table 1). There is a diverse range of chemical groups on the VOCs, including alcohol (~ 30%), aldehyde, alkane, ketones (~ 10–15% for each), and terpene (~ 5%) functional groups being the most common. Of these 84 VOCs, none was is were 4-ethyl-2-methoxyphenol (six species), 3

dentified from all 11 of the species, and the VOCs that were detected in the most species were 4-ethyl-2-methoxyphenol (six species), 3-undecen-2-one (six species), 1-octen-3-ol (five species), and 1-decanol (five species). Approximately half of the VOCs were only identified from one of the species, supporting the diversity of VOCs that can be produced by *Pythium* or *Phytophthora* species.

**Table 1:** Inventory of volatile organic compounds produced by *Pythium* or *Phytophthora* species sorted in order of increasing PubChem CID number. The information presented here is a subset of the information in Table S1A

PubChem CID	Name(s) of compound (alternative names separated by a semicolon)	Chemical formula	*Ph. cactorum* (Loulier et al. 2020)	*Ph. cambivora* (Sherwood et al. 2024)	*Ph. cinnamomi* (Qiu et al. 2014)	*Ph. citricola* (Sherwood et al. 2024)	*Ph. gonapodyides* (Sherwood et al. 2024)	*Ph. multivora* (Sherwood et al. 2024)	*Ph. plurivora* (Sherwood et al. 2024)	*Ph. polonica* (Sherwood et al. 2024)	*Ph. ramorum* (Loulier et al. 2020)	*Ph. syringae* (Sherwood et al. 2024)	*Py. oligandrum* (Sheikh et al. 2023b)
179	Acetoin; 3-hydroxy-2-butanone; 2-Butanone, 3-hydroxy-	C_4_H_8_O_2_			X				X				
180	Acetone	C_3_H_6_O	X						X				
262	2,3-Butanediol	C_4_H_10_O_2_						X					
332	2-Methoxy-4-vinylphenol	C_9_H_10_O_2_			X								
454	1-Octanal	C_8_H_16_O											X
702	Ethanol	C_2_H_6_O			X						X		
957	1-Octanol	C_8_H_18_O	X										
2682	1-Hexadecanol	C_16_H_34_O						X					
2969	Decanoic acid	C_10_H_20_O_2_		X			X		X				
3893	Dodecanoic acid	C_12_H_24_O_2_		X			X	X					
6054	2-Phenylethanol; Phenylethyl alcohol	C_8_H_10_O			X						X		X
6184	Hexanal	C_6_H_12_O		X	X		X			X			
6569	2-Butanone	C_4_H_8_O			X								
6654	α-Pinene	C_10_H_16_							X				X
7302	Butyrolactone	C_4_H_6_O_2_			X								
7361	2-Furanmethanol	C_5_H_6_O_2_										X	
7461	γ-Terpinene	C_10_H_16_											X
7895	2-Pentanone	C_5_H_10_O			X								
8048	Ethyl decanoate	C_12_H_24_O_2_											X
8051	2-Heptanone	C_7_H_14_O											X
8058	Hexane	C_6_H_14_			X								
8103	1-Hexanol; Hexanol	C_6_H_14_O	X						X				
8129	1-Heptanol	C_7_H_16_O	X					X					
8163	2-Undecanone	C_11_H_22_O											X
8174	1-Decanol	C_10_H_22_O		X			X	X	X	X			
8182	Dodecane	C_12_H_26_											X
8207	1-Tridecanol	C_13_H_28_O						X					
8209	1-Tetradecanol	C_14_H_30_O		X				X					
8723	2-Methyl-butanol	C_5_H_12_O									X		
8914	1-Nonanol	C_9_H_20_O		X			X	X		X			
9862	Methyl heptenone	C_8_H_14_O											X
10413	4-Hydroxy-butanoic acid	C_4_H_8_O_3_							X				
10911	Dodecamethylcyclohexasiloxane	C_12_H_36_O_6_Si_6_			X								
10914	Hexamethylcyclotrisiloxane	C_6_H_18_O_3_Si_3_			X								
11006	Hexadecane	C_16_H_34_											X
11196	2,6-Nonadienal	C_9_H_14_O		X			X						
11463	Terpinoline	C_10_H_16_											X
11509	3-Hexanone	C_6_H_12_O											X
12232	Dimethyl disulfide	C_2_H_6_S_2_	X		X								
12367	2-Nonanol	C_9_H_20_O						X	X				
12389	Tetradecane	C_14_H_30_											X
12399	Dioctyl ether	C_16_H_34_O											X
12844	δ-Dodecalactone	C_12_H_22_O_2_		X			X						
13190	Undecene	C_11_H_22_											X
14257	Undecane	C_11_H_24_											X
15448	2-Undecanol	C_11_H_24_O			X								
15449	2-Tridecanol	C_13_H_28_O						X					
16821	γ-Dodecalactone	C_12_H_22_O_2_			X		X	X		X			
18827	1-Octen-3-ol	C_8_H_16_O	X		X	X				X	X		
19310	Dimethyl trisulfide	C_2_H_6_S_3_			X								
20283	Cyclohexane, hexyl-	C_12_H_24_											X
20745	DL-6-methyl-5-hepten-2-ol	C_8_H_16_O			X								
22311	Limonene	C_10_H_16_											X
26049	∆-3-Carene	C_10_H_16_							X				
31242	4-ethylphenol; Phenol, 4-ethyl-	C_8_H_10_O			X				X				
31260	3-Methyl-butanol; 1-Butanol, 3-methyl-	C_5_H_12_O									X		
33191	1-Phenyl-2-hexanone	C_12_H_16_O						X					
62465	4-Ethyl-2-methoxy-phenol; Phenol, 4-ethyl-2-methoxy-; 4-Ethylguaiacol	C_9_H_12_O_2_		X	X	X		X	X	X			
66310	3-Nonen-1-ol	C_9_H_18_O								X			
79022	Tricyclo[2.2.1.0(2,6)]heptane, 1,3,3-trimethyl-	C_10_H_16_											X
92651	4-Decen-1-ol	C_10_H_20_O								X			
93071	5-Methyldecane; Decane, 5-methyl-	C_11_H_24_			X								
103851	5,9-Undecadien-2-ol, 6,10-dimethyl-	C_13_H_24_O			X								
137658	2,4,6-Trimethylheptane	C_10_H_22_			X								
161533	Aristolochene	C_15_H_24_			X								
246728	3-Octanone	C_8_H_16_O	X								X		X
519982	Methyl 2,4,6-trimethyl benzoate	C_11_H_14_O_2_			X								
545608	Decane, 2,6,8-trimethyl-	C_13_H_28_											X
1715061	5-Methyl-3-heptanon	C_8_H_16_O			X								
5283321	2,4-Heptadienal	C_7_H_10_O		X			X		X				
5283339	2,4-Nonadienal	C_9_H_14_O		X			X			X			
5283349	2,4-Decadienal (E,E)-	C_10_H_16_O		X			X			X			
5283356	2-Undecenal	C_11_H_20_O								X			
5318599	2-Octen-1-ol	C_8_H_16_O	X										
5352876	3,5-Octadien-2-one	C_8_H_12_O		X			X						
5362760	2,4-Undecadien-1-ol	C_11_H_20_O								X			
5365758	1,5-Dimethyl cyclooctadiene	C_10_H_16_											X
5366074	beta-Damascenone	C_13_H_18_O			X								
5463933	2,6-Dodecadienal	C_12_H_20_O					X						
6427087	2,4-Decadienal (E,Z)-	C_10_H_16_O		X			X	X		X			
6437801	3-Undecen-2-one	C_11_H_20_O		X	X		X	X	X	X			
6553875	Pinanediol	C_10_H_18_O_2_											X
11217478	6-Undecen-2-one	C_11_H_20_O			X	X				X		X	
56936219	6-Pentadecen-2-one	C_15_H_28_O						X					

*Ph. cinnamomi* is the most extensively studied species, with three studies identifying VOCs from *Ph. cinnamomi* with either 21 VOCs (Qiu et al. 2014), three VOCs (Loulier et al. 2020), or seven VOCs (Sherwood et al. 2024) identified. The VOC 4-ethyl-2-methoxyphenol was identified in all three studies, and 1-octen-3-ol was identified in the studies of Qiu et al. (2014) and Loulier et al. (2020) but no other *Ph. cinnamomi* VOCs were identified in more than one of the three studies. Two studies identified VOCs from *Ph. plurivora*, and the six VOCs identified from a culture on potato dextrose medium (Loulier et al. 2020) were all different from the seven VOCs identified on Elliott’s medium (Sherwood et al. 2024). For the other species where there is only a single study that analyzed the VOCs, the species followed by the number of VOCs identified in parenthesis are listed: *Ph. cactorum* (eight VOCs), *Ph. ramorum* (six VOCs) (Loulier et al. 2020), *Py. oligandrum* (23 VOCs) (Sheikh et al. 2023b), *Ph. cambivora* (15 VOCs), *Ph. citricola* (three VOCs), *Ph. gonapodyides* (15 VOCs), *Ph. multivora* (16 VOCs), *Ph. polonica* (15 VOCs), and *Ph. syringae* (two VOCs) (Sherwood et al. 2024). *Py. oligandrum* differs from all the *Phytophthora* species insofar as it is not plant pathogenic and instead a plant beneficial species. Of the 23 VOCs identified from *Py. oligandrum*, three VOCs (α-pinene, 3-octanone, and 2-phenylethanol) were identified from at least one of the *Phytophthora* species, and the other 20 VOCs were only identified from *Py. oligandrum*.

### Comparison with identified VOCs produced by other microorganisms

For the 84 VOCs identified from *Pythium* or *Phytophthora* species, 58 had an entry as either bacterial- or fungal-produced VOC in the microbial VOC 3.0 (mVOC) database (Lemfack et al. 2018). Some of these mVOCs entries were mainly from bacteria (e.g., 2-Undecanone), others mostly from fungi (e.g., 1-Octen-3-ol), and others with similar proportions of entries from fungi and bacteria (e.g., dodecane) (Table S1B). Note that when searching the mVOC database, there were no entries for oomycetes or other stramenopiles (heterokonts) that oomycetes are phylogenetically more closely related to than fungi, although later updates of the mVOC database may include oomycete entries. Recently, the literature on volatile compounds produced by edible macroalgae was reviewed (Li et al. 2023). For the 84 VOCs identified from *Pythium* or *Phytophthora* species, at least 26 were also produced by a least one of the seven edible macroalgal species reviewed (3-hexanone, α-pinene, 3-octanone, limonene, undecane, tetradecane, acetone, 1-octen-3-ol, 2-octen-1-ol, 1-octanol, butyrolactone, 2-butanone, ethanol, 2-pentanone, hexanal, 2,6-nonadienal, decanoic acid, 3,5-octadien-2-one, 2,3-butanediol, 1-heptanol, 2-heptanone, 1-hexanol, 1-octanal, 2,4-heptadienal, 2,4-decadienal, and 3-methyl-butanol) (Li et al. 2023).

In a previous study, a list was compiled of microbial VOCs that were shown in the literature in vitro and/or in vivo as bioactive inhibitors of phytopathogens (Almeida et al. 2022). The oomycete VOCs in Table 1 were compared with those VOCs produced by bacteria and fungi, summarized by Almeida et al. (2022). In an analysis of the 84 VOCs identified from *Pythium* or *Phytophthora* species with the VOCs listed by Almeida et al. (2022), 32 of the VOCs were listed by Almeida et al. (2022) as having biocontrol-related properties towards one or more plant pathogens. This large overlap highlights the potential biological activities of the oomycete-produced VOCs that may function in microbe-microbe interaction in natural settings, such as the VOCs produced by the oomycete plant pathogens, and also the potential for oomycete biocontrol agents that produce these VOCs to control crop diseases.

## The physiological effects of *Pythium* and *Phytophthora*-produced VOCs

VOCs produced by *Pythium* and *Phytophthora* species can influence the physiology of microorganisms and plants, resulting in beneficial or harmful effects on these microorganisms and plants and, perhaps, overall ecosystem functioning (Fig. 1). The physiological effects of fungal VOCs were comprehensively reviewed recently by El Jaddaoui et al. (2023), highlighting the diverse impacts VOCs can have on microbial and plant systems.

### Physiological effects of *Pythium* and *Phytophthora*-produced VOCs on microorganisms

The physiological effects of VOCs on microorganisms are wide-ranging and can include the inhibition of microbial growth. The overall impact of VOCs and the specific effects of identified VOCs on various microorganisms have been studied. *Py. oligandrum* VOCs have been shown to inhibit the growth of four plant pathogens. The *Py. oligandrum* lMI 133857 strain inhibited the growth of *Ascochyta medicaginicola* (syn. *Phoma medicaginis*) (Bradshaw-Smith et al. 1991)*.* The *Py. oligandrum* strain El-U1122 inhibited the growth of *Fusarium oxysporum and Py. ultimum* (El-Katatny et al. 2006), and the *Py. oligandrum* GAQ1 strain inhibited the growth of *Py. myriotylum* (Sheikh et al. 2023b). The inhibitory effects of *Py. oligandrum* GAQ1 VOCs on mycelial growth persisted even after exposure to *Py. oligandrum* VOCs was discontinued (Sheikh et al. 2023b). In contrast, the volatile compounds from the strain of *Py. oligandrum* contained in Polyversum® did not inhibit the radial colony growth of *Fusarium graminearum*, but the VOCs did reduce mycotoxin production by > 50% (Pellan et al. 2021). The physiological effects of individual VOCs identified from the *Py. oligandrum* GAQ1 volatilome on *Py. myriotylum* growth were studied. The individual *Py. oligandrum* GAQ1 VOCs methyl heptenone, limonene, 2-undecanone, and 1-octanal showed the strongest inhibition of *Py. myriotylum* mycelial growth*.* Additionally, exposure of *Py. myriotylum* hyphae to these individual VOCs, as well as the total *Py. oligandrum* VOCs, led to an increase in reactive oxygen species (ROS) levels in the hyphae (Sheikh et al. 2023b).

While individual VOCs from *Phytophthora* species have been characterized, the effects of total *Phytophthora* VOCs on microorganisms have not been investigated in the way that the growth inhibitory effects of total *Py. oligandrum* VOCs were investigated. Therefore, microbial growth inhibitory properties of *Phytophthora* VOCs are inferred from the reported effects of individual VOCs produced by *Phytophthora* species. It is useful to speculate that the *Phytophthora* VOCs may inhibit other potentially plant-beneficial microorganisms in the soil in competitive interactions within the soil microbiota and, therefore, contribute indirectly to the infection of plant hosts. Some of the most commonly identified VOCs produced by *Phytophthora* include 1-octen-3-ol, 3-octanone, 2-undecanone, and decanoic acid (Qiu et al. 2014; Loulier et al. 2020; Sherwood et al. 2024) (Table S1A). The VOC 3-Octanone has been reported to inhibit the mycelial growth of the oomycete *Py. myriotylum* (Sheikh et al. 2023b)*.* The VOC 2-undecanone has been reported as toxic to the nematode *Meloidogyne incognita* (Huang et al. 2010) and also inhibited the growth of the fungus *Sclerotinia sclerotiorum* (Massawe et al. 2018). Recently, decanoic acid has been reported to be produced by the *Phytophthora* species, *Ph. plurivora*, *Ph. gonapodyides*, and* Ph. cambivora* (Sherwood et al. 2024), and a previous study showed that decanoic acid can inhibit fungal growth (Guo et al. 2019)*.* More broadly, the VOCs produced by *Phytophthora* species could also influence bacterial communities such as by inhibiting biofilm formation and quorum sensing, as described by Garbeva et al. (2014) on the effects of bacterial VOCs on bacterial communities.

It should be noted that none of the four primary research articles reviewed and summarized in Table 1 measured the concentration of individual VOCs in rhizosphere soil inoculated with the *Phytophthora* or *Pythium* species that were reported to produce VOCs. Therefore, there is a large degree of speculation regarding the effect of the produced VOCs on the inhibition of other microorganisms in rhizosphere soils, particularly considering that the production of some VOCs is medium-dependent, e.g., where the VOC is a breakdown product of substrates found in common laboratory media that may not be found in rhizosphere soils.

### Physiological effects of *Pythium* and *Phytophthora*-produced VOCs on plants

*Py. oligandrum* GAQ1 VOCs can have substantial effects on plant physiology, particularly in terms of growth promotion. The VOCs emitted by *Py. oligandrum* GAQ1 can promote the growth of *Nicotiana benthamiana* and ginger by increasing root and shoot growth (Sheikh et al. 2023a). Individual VOCs produced by *Py. oligandrum* GAQ1 can promote *N. benthamiana* growth, e.g., 3-octanone and hexadecane can contribute to *N. benthamiana* growth by increasing the biomass and modulating of hormone signaling may be involved (Sheikh et al. 2023b). Notably, other VOCs produced by *Py. oligandrum* GAQ1 neither inhibited nor promoted *N. benthamiana* seedling growth (Sheikh et al. 2023b). One of these VOCs, 2-phenylethanol, has been shown to inhibit the growth of *Arabidopsis thaliana* (Wenke et al. 2012) and alfalfa (Ulloa-Benítez et al. 2016) at relatively high concentrations. However, it was noted that the inhibitory concentrations of 2-phenylethanol were unlikely to be found in natural settings (Garbeva and Weisskopf 2020). Notably, of the two *Py. oligandrum* GAQ1 VOCs (3-octanone and hexadecane) that promoted the growth of *N. benthamiana*, hexadecane was not identified in any of the *Phytophthora* species (Table 1). Hexadecane may potentially be a VOC associated with plant-beneficial oomycetes and not plant-pathogenic oomycetes, but far more sampling of *Pythium* species is required as, to date, there are no reports of any VOC identification from plant-pathogenic *Pythium* species.

*Phytophthora* species generally have a plant-pathogenic lifestyle (Brasier et al. 2022), and some of the VOCs produced by *Phytophthora* species may be potential virulence factors and contribute to causing the disease. The VOC 1-octen-3-ol is produced by several *Phytophthora* species, and it has been shown to induce an oxidative burst in leaves and shorten the roots in *A. thaliana*, indicating a phytotoxic effect (Splivallo et al. 2007). Similarly, methyl-butanol (2-methyl-1-butanol) is produced by *Ph. ramorum*, and methyl-butanol has been characterized previously as inhibitory of tomato seed germination and root elongation (Sánchez-Ortiz et al. 2016). The VOCs that could potentially be virulence factors could also have a biotechnological application in aiding disease detection because detecting VOCs that are virulence factors as opposed to other VOCs means that a disease-causing strain is being detected.

## Applications of *Pythium* and *Phytophthora*-produced VOCs

### Biocontrol using *Pythium* and *Phytophthora* species

Biological control can be broadly defined as the use of living organisms (biocontrol agents) to combat pathogens, pests, and weeds (Stenberg et al. 2021). If the biocontrol agent can produce VOCs, then depending on the VOCs, they have the potential to contribute to combatting pathogens, pests, and weeds, e.g., biocontrol of plant diseases using VOCs represents a promising approach in agriculture and was reviewed recently by Tilocca et al. (2020). The VOCs can contribute to the biocontrol of plant diseases by one or more mechanisms, such as by inhibiting the growth of plant pathogens and priming plant defense responses (Tilocca et al. 2020).

The *Py. oligandrum* GAQ1 strain, a potential biocontrol agent, has been demonstrated to produce dozens of VOCs (Table 1). The *Py. oligandrum* GAQ1 strain can control *Pythium* soft-rot disease of ginger caused by *Py. myriotylum* (Daly et al. 2021), and two recent studies investigated the contribution of *Py. oligandrum* GAQ1-produced VOCs to the control of soft-rot disease of ginger (Sheikh et al. 2023b, 2023a). The total *Py. oligandrum*-produced VOCs inhibited mycelial growth of *Py. myriotylum* in a bi-partite plate, and VOC-pre-exposed inoculum of *Py. myriotylum* had smaller disease lesions on detached ginger leaves indicating that the growth-inhibitory effect of VOCs could be contributing to the overall disease control effect of *Py. oligandrum* (Sheikh et al. 2023b). The total *Py. oligandrum*-produced VOCs led to increased plant growth using a pot-jar assembly to expose ginger plants to VOCs, indicating that growth promotion via *Py. oligandrum*-produced VOCs could also be contributing to the disease control effect (Sheikh et al. 2023a). To make more general inferences about the contribution of *Py. oligandrum*-produced VOCs to disease control, it is essential to identify the VOCs in the *Py. oligandrum* strains used in commercial biocontrol products such as Polyversum® (Kurzawińska and Mazur 2007; Pellan et al. 2021; Pisarčik et al. 2022) to add more weight to the claims of practical application in agriculture of *Py. oligandrum*-produced VOCs. Also, it is noteworthy that although there is good evidence that *Py. oligandrum* GAQ1-produced VOCs contribute to the disease control effect on ginger, there are also other disease control mechanisms that likely have a greater contribution than VOCs of *Py. oligandrum*, such as (myco-)parasitism (see recent review of Bělonožníková et al. (2022)) and plant-hormone auxin production (Le Floch et al. 2003), and plant defense elicitor production (Benhamou et al. 2001).

Despite the potential benefits, there are limitations and challenges in fully exploiting VOCs for biocontrol, including issues related to delivery methods such as diffusion of the VOCs to below bioactive concentrations. In cropping systems where plastic mulch is used, the mulch could help reduce the diffusion of VOCs from the soil, thereby facilitating the inhibitory and plant growth-enhancing effects of *Py. oligandrum*-produced VOCs. There may also be air pockets within the soil that may facilitate the accumulation of higher concentrations of VOCs to inhibit the plant pathogens in these soil air pockets. Exploiting VOC-producing *Pythium* biocontrol strains may be more feasible in nurseries and glasshouses than in field conditions.

As well as applications related to the biocontrol of plant pathogens, the plant pathogens themselves can have applications in the biocontrol of weeds. A commercial product called DeVine® used *Phytophthora palmivora* as a bioherbicide to control vines that grow around citrus trees (Kenney 1986), and a later review reported that DeVine® is no longer commercially available (Bailey 2014). There are no reports of *Ph. palmivora* producing VOCs, but *Ph. palmivora* highlights the potential of *Phytophthora* plant pathogens to control weeds, and the reports of various *Phytophthora* plant pathogens producing VOCs (Table 1) that have phytotoxic properties could lead to phytotoxic VOCs contributing to the control of weeds. However, the broad host-range of *Phytophthora* plant pathogens may increase the stringency of regulatory approval for biocontrol products where *Phytophthora* is the active agent.

### Use of *Pythium* and *Phytophthora*-produced VOCs to detect and discriminate pathogens in planta

Cost-effective and accurate detection of *Pythium* and *Phytophthora* plant pathogens is important, and VOCs hold promise for enabling both economical and accurate pathogen detection. Alongside existing DNA-based detection technologies in diagnostic assay development, using VOCs is an emerging technology (Geiser et al. 2023). VOCs can be used to detect both plant disease and plant pathogen, and this is often done by detecting VOCs that are likely produced by either the infected plant or constitutively produced or infection-induced in the plant pathogen. Numerous examples of fungal- and bacterial-caused diseases are detected via volatiles; see the list from the review by Wilson (2018), but there are fewer examples for *Pythium-* and *Phytophthora*-caused diseases.

VOCs detected in plant interaction with *Phytophthora* species demonstrate the potential for *Phytophthora*-produced VOCs to aid disease or pathogen detection. In a study of VOCs produced when *Ph. cinamomi* infected lupin seedlings, half of the 16 VOCs identified from the infected seedlings were also identified from a monoculture of *Ph. cinamomi* (Qiu et al. 2014). This data supports using VOCs from a *Phytophthora* species to indicate the presence of that *Phytophthora* species in an infected plant. A limitation of these studies with VOCs is that they sometimes lack an oomycete-only control to compare with the oomycete-inoculated plant sample. In *Ph. ramorum-*infected rhododendron, there were 32 VOCs identified, and while most are likely plant-produced VOCs, 1-octen-3-ol was also detected (McCartney et al. 2018). The 1-octen-3-ol VOC may be produced by *Ph. ramorum* because another study showed that 1-octen-3-ol was produced from a *Ph. ramorum* monoculture (Loulier et al. 2020). In *Ph. infestans-*infected potato tubers, 28 VOCs were identified (De Lacy Costello et al. 2001), and it is not possible to speculate whether any of these VOCs might be produced by *Ph. infestans* because, to our knowledge, there are no reports of identification of VOCs from a *Ph. infestans* monoculture. Recently, 14 volatile compounds were identified that could distinguish between eight *Phytophthora* species in vitro, but in a preliminary analysis of VOCs from infected trees, other VOCs that likely were of plant origin were detected (Sherwood et al. 2024).

Key technologies for the in-field or on-site detection of volatiles include portable GC–MC devices, electronic nose (e-nose) devices, and smartphone-based VOC sensors (Tholl et al. 2021). Using an e-nose, *Ph. plurivora* volatile compounds were detected from a monoculture (Borowik et al. 2022), and the e-nose could differentiate between *Py. intermedium* and *Ph. plurivora* (Borowik et al. 2021). This result may support an application in the detection of pathogens from contaminated potting soil, but more data is needed to support the discrimination from infections in planta. An alternative to e-nose for detecting volatiles from *Phytophthora* species is using trained dogs as a real-time mobile technology to detect the volatile scent from *Phytophthora* species. As reviewed previously, dogs can distinguish different scents from biological sources (Angle et al. 2016). In a recent study, trained dogs could detect the scent from *Ph. agathidicida*-inoculated oats with a sensitivity of ~ 70% and a precision of ~ 50%, whereas other off-target control scents of two other *Phytophthora* species (*Ph. cinnamomi* and *Ph. multivora*) were sometimes detected (Carter et al. 2023). The results from the *Ph. agathidicida* scent detection by dogs supported their use as a screening-type detection tool that likely needed confirmation with a more sensitive and precise method. In another study, an ecological scent detection dog identified four *Phytophthora* species (*Ph. nemorosa*, *Ph. ramorum*, *Ph. cactorum*, and *Ph. cinnamomi*) by scent from soil samples and drained water with 100% accuracy, although the authors noted the preliminary status of the results (Swiecki et al. 2018).

A critical feature of plant pathogen discrimination is identifying uniquely produced VOCs, and VOC analysis of more species in the future is important for this, as well as reporting of negative findings where no or few VOCs were detected from a *Pythium* or *Phytophthora* species. VOCs from beneficial *Pythium* species and plant pathogenic species need to be distinguished to avoid discarding healthy plant material from a nursery that contains beneficial *Py. oligandrum* isolates, and the 23 VOCs reported as produced by *Py. oligandrum* can guide this task, but VOC data from other plant beneficial *Pythium* species is also required. As well as identifying unique VOCs, the unique ratios of commonly produced VOCs may also be diagnostic of plant pathogens in particular contexts. It is also worth noting that in wet or aquatic environments such as hydroponic growth conditions, there will likely be other water molds or stramenopiles such as algae present, and these other stramenopiles could potentially produce the same volatile compounds as *Pythium* or *Phytophthora* plant pathogens thus leading to false positive detections. As described in the previous section on volatile production, there was an overlap between the VOCs produced by *Pythium* and *Phytophthora* species and those produced by edible macroalgae (Li et al. 2023).

## Emerging technological trends and opportunities for further research

The metabolic origin of *Pythium-* and *Phytophthora-produced* VOCs is an area for future research. In a recent review, true fungal VOCs are described as degradation products of fatty acids, biotransformation of amino acids, or breakdown products of substrates the fungus is growing on (Inamdar et al. 2020). The likely metabolic origin of *Pythium* and *Phytophthora* VOCs is likely similar and varied as that of true fungi, except that biosynthetic gene clusters are unlikely to be a major metabolic origin of *Pythium* and *Phytophthora* VOCs as there are fewer biosynthetic gene clusters in *Pythium* and *Phytophthora* genomes. One possible way to distinguish mainly de novo metabolically synthesized VOCs from breakdown products of substrates is to use stable isotope labeling. In a study with bacterial VOCs, stable isotope labeling was used to identify actively produced bacterial VOCs from animal-associated samples (Phan et al. 2022). One of the drawbacks of conventional mass spectrometry related to VOCs and metabolic origin is that D- and L enantiomers cannot be distinguished. A new method can determine the enantiomeric ratios of a compound by mass spectrometry alone, which has the potential to more readily indicate the enantiomers of VOCs (Zhou et al. 2024).

A drawback of data on VOCs from *Pythium* and *Phytophthora* species is that it is generally qualitative or semi-quantitative, partly because the commonly used solid-phase microextraction (SPME) method has a limited quantitative range (Nolvachai et al. 2023). A platinum catalyst and proton transfer mass spectrometry are effective in quantifying total VOCs (albeit not individual VOCs), whereby headspace VOCs are oxidized to CO_2_ (Schoen et al. 2016). Recently, studies with *Trichoderma* species used proton transfer reaction time-of-flight mass spectrometry (PTR-ToF–MS) to quantify volatiles in real-time (Lochmann et al. 2024) and to develop a VOC-based chemotyping platform which also used machine learning in the data analysis (Guo et al. 2020). The higher throughput of some of these analysis methods could facilitate the analysis of more biocontrol agents from the *Pythium* genus, such as *Py. periplocum* (Paul 1999; Liang et al. 2020) and *Py. guiyangense* (Shen et al. 2019).

One of the key challenges with the use of antimicrobial compounds is the potential for the development of resistance to the compound (R4P-Network 2016). Analysis of how the VOC-producing *Pythium* biocontrol agent species are resistant to the toxic effects of their own VOCs could hint at resistance mechanisms to these VOCs and better inform which VOCs are less likely to have resistance developed against them in field conditions. Also, exposing the target plant pathogens to VOCs produced by the *Pythium* biocontrol agent and studying the responses of the plant pathogen (e.g., expression of de-toxifying enzymes) could hint at potential resistance mechanisms. A recently developed method could be useful for this analysis because it facilitates the unidirectional exposure of a microbe to the VOCs of another microbe (Bruisson et al. 2023).

## Conclusion

VOCs produced by *Pythium* and *Phytophthora* species are a promising area of future research for biotechnological applications in disease control from the perspectives of both a biocontrol agent and in disease detection.

## Supplementary information

Below is the link to the electronic supplementary material.

Supplementary file1 (XLSX 44.3 KB)
